# Toward a Theory of the Primo Vascular System: A Hypothetical Circulatory System at the Subcellular Level

**DOI:** 10.1155/2013/961957

**Published:** 2013-06-18

**Authors:** Byung-Cheon Lee, Ji Woong Yoon, Sang Hyun Park, Seung Zhoo Yoon

**Affiliations:** ^1^Ki Primo Research Laboratory, Division of Electrical Engineering, KAIST Institute for Information Technology Convergence, Korea Advanced Institute of Science and Technology (KAIST), Daejeon 305-701, Republic of Korea; ^2^Impedance Imaging Research Center and Department of Public Administration, College of Politics and Economics, Kyung Hee University, No. 1 Hoegi-dong Dongdaemun-gu, Seoul 130-701, Republic of Korea; ^3^Department of Anesthesiology and Pain Medicine, College of Medicine, Korea University, Seoul 136-705, Republic of Korea

## Abstract

This paper suggests a theoretical framework for the primo vascular system (PVS), a hypothetical circulatory system, in which extracellular DNA microvesicles interact to form and break down cell structures. Since Bonghan Kim reported the existence of Bonghan ducts and the SNU research team reinvestigated and named it the PVS, there has been series of studies trying to examine its structure and functions. In this paper, we hypothesize that the PVS is the network system in which extracellular DNA microvesicles circulate and interact at the subcellular level, forming and breaking down cell structures. This idea integrates A. Béchamp's idea of microzymas and Bonghan Kim's idea of sanals. A proof of this idea may complement modern medical theory, perhaps providing an essential clue for an alternative solution dealing with modern healthcare problem.

## 1. Introduction

Since the Bonghan ducts were identified by Bonghan Kim in the 1960s [1–4] and renamed as Primo vascular system (PVS) by the Seoul National University (SNU) research group in 2002, the PVS has been studied by various research groups, presuming it as a new anatomic system, apart from blood and lymphatic systems [5]. The PVS has been found in various organs, and a variety of possible functions of the PVS have been proposed, based on the elements found within the PVS. The liquid carried within the PVS consists of various microparticles, such as DNA, proteins, and hormones [5]. However, the studies on the PVS are at the primitive stage showing elements flowing with the PVS and basic structures of the PVS. And it is rare to see a comprehensive discussion on a theoretical framework or approach of the PVS, trying to understand where it can stand with the modern medical theories.

Hence, this paper provides a brief overview of the PVS research up to date. And the hypothetical functions of the PVS are proposed, which may help building a theoretical framework at the subcellular level activities in the living beings. The main idea is that the PVS is a circulatory system in which microparticles, such as extracellular DNA (eDNA) microvesicles, are floating and interacting [6, 7]. Moreover, the PVS works as the primordial physical blueprint of living organisms [8], which enables eDNA to generate, and degenerate cell-like structures [9]. 

These hypothetical functions of the PVS are established by matching the sporadic ideas from the previous literatures at the subcellular level, which are ignored for decades. We expect to see researches that prove the hypotheses proposed in this paper in the near future. This may enable a further understanding of a new biological structure and the functions of the living beings. Furthermore, we hope that the PVS, as a primordial anatomical blueprint for living beings, can provide an eye-opening paradigm shift in the modern medical theory that treats chronic diseases, such as cancer and Alzheimer's disease. 

## 2. What We Know about the PVS

In the 1960s, Bonghan Kim urged that he identified a new thread-like circulatory system and called that the Bonghan ducts, which is now renamed as the PVS by the SNU research group [1, 5]. Although he mainly studied rabbits and mice, Bonghan Kim mentioned that the PVS can be observed in various other mammalian species [2, 3]. He also reports that sanals flow within PVS, and sanals combine with one another, forming cells [4]. His research was suddenly shut down and he disappeared from the modern medical history, although his initial works caught some intentions from other countries [5, 6].

After several decades, to reawaken and confirm Bonghan Kim's discovery, SNU research group reinvestigated the PVS mostly in small animals since 2002. The major achievement of the group is the identification and observation of the PVS in various organs of different kinds of animals, and the advancement of technical methods for identifying the PVS. For example, the PVS was observed in the lymphatic vessels of rabbits [10, 11], in the brains and spinal cords of rabbits [12] and in the brains of rats [13]. Moreover, for the first time, the PVSs freely floating in bovine heart chambers were observed [14]. Since then, additional cases have been reported for cows [15] and dogs [16].

The structure of the PVS has been examined in more details. The PVS consists of the primo nodes (Bonghan corpuscles) and the primo vessels (Bonghan ducts). The PVS exists not only in the skin, but also is widely distributed throughout the internal organs. The PVS is classified into two parts: intravascular and extravascular. The intravascular primo vessel runs inside the blood vessel or the lymphatic vessel, while the extravascular primo vessel runs outside the vessel [10, 12, 17]. Although the intravascular primo vessel and the extravascular primo vessel take different directions from each other, there is no difference between them in structure, both having a peculiar rod shape [18].

In addition, the primo vessel comprises bundles of primo lumens. The primo lumen is very soft and has a thin wall, which consists of endothelial cells of a single layer [1]. The contents of the primo lumen often appear as granules when they are stained by a routine method. Moreover, it has been established, by cytochemical reaction, that they contain DNA vesicles [19]. The PVS has also been observed to have a web-like network structure with primo nodes connecting primo vessels, although it is not known to what extent the PVS is networked and whether that network is limited to certain organs [16, 17].

## 3. Hypothetical Functions of the PVS

### 3.1. A Third Circulatory System

The PVS is hypothesized as a third circulatory system. Various basic liquids flow within the PVS. A liquid carried by the PVS was found to be rich in basophilic granules, which can be observed individually, as well as in clusters [20]. Breaking the liquid down into its components, proteins [21], stem cell niches [22], microcells [23, 24], and hormones are identified to flow with the PVS [25, 26]. Hence, the PVS seems to function as a circulatory system conveying variety of elements which influence the whole living being.

The flow speed of the liquids in the PVS has been measured [27]. By injecting alcian blue dye in to the PVS directly, the flow speed was measured to be up to 0.3 mm/s [28] with a range of 100–800 *μ*m/second when directly measured using radioactive tracers [29, 30]. These values are significantly higher than those measured in lymphatic vessels [20]. This supports Bonghan Kim's idea that speed of primo fluid movement depends upon various morphological features [31].

### 3.2. A Biological Blueprint and Channel

More specific functions of the PVS are suggested by analyzing the contents of the liquids flowing within the PVS. We hypothesize that the PVS may be a primordial circulatory system that works as a blueprint for developing and repairing the vessels and organs [32]. 

The PVS in the vitelline membrane in eggs has been observed within 16–24 hours of incubation, and the putative PVS clearly developed earlier than the formation of the extra-embryonic vessels, letting alone the establishment of the heart and intramembranous vessels [8]. Moreover, the primo vessels are surrounded by a membrane. The membrane has a high concentration of hyaluronic acid, and it is reported that hyaluron is responsible for cell growth and differentiation [33].

An additional function posited for the PVS is as an optical channel of biophoton emission, because collagen is involved in the photon-emitting processes. This raises the possibility that the biophotons may be the electromagnetic signals that play a key role in the processes of cell development and differentiation, or else the DNA may act as a photon store and coherent radiator [34].

### 3.3. A Network for the Fusion and Diversion of eDNA Microvesicles

The last hypothetical function of the PVS that we propose is that the PVS may allow eDNA microvesicles to systematically interact with one another, forming cell-like structures [7]. This function may be linked with immune system functions [35]. These activities of eDNA microvesicles at the subcellular level imply that a generation or degeneration of a cell structure may be a ramification of fusion and division at the subcellular level of the PVS. 

This hypothesis was developed through several steps. First, Lee et al. attempted to demonstrate that a sanal exists and has DNA inside and grows by a budding process [36]. Then Lee et al. [37] observed about 1 *μ*m sized extracellular vesicles, which seem quite similar to what Bonghan Kim reported in his paper [4], that cell-like structures with DNA signal in a concentric growth pattern grew in fertilized eggs [37]. In order to observe the fusion of eDNA microvesicles, Lee et al. developed a special incubating chamber equipped with a microscope, for simultaneous incubation and observation of extracellular DNA vesicles [37]. 

Surprisingly, it seems that this fusion of eDNA microvesicles is closely associated with recent research in the field of microvesicles, sometimes called exosomes or microparticles [38]. Microvesicles have been shown to play a role in intercellular communication and regeneration of tissues [39]. However, studies on microvesicles do not mention systematic flow of the microvesicles nor any circulation of microvesicles. The studies are now at the level of defining the anatomical structure and basic functions of microvesicles, which are especially associated with cancer tumors [40]. 

## 4. PVS: A Network System for the Subcellular Level Activities 

Building on the third hypothesis proposed in the previous section, the PVS is proposed to be a network system where fusion of eDNA vesicles occurs, which eventually form cell-like structures [7]. Observation of this phenomenon implies that the PVS could be a system allowing the interaction of eDNA vesicles to form and break down cell structures in various patterns at the subcellular level, conditional on the biological environment.

This idea of subcellular level activities in the living organ actually can be traced back to the idea of pleomorphism, which is anatomically and physiologically different from monomorphism, the modern orthodox medical paradigm. Pleomorphism, focusing on the basic living element at the subcellular level, was introduced in the mid-19th century by Béchamp, a French scientist [41]. Béchamp's idea was that the *microzyma* (*a small special class of immortal enzymes*) is the smallest living element that forms, granulates, and degrades the living cells and organs. He also showed that these microzymas were found to be present in all things, whether living or dead, and they persist even when the host dies [32]. Béchamp's idea of microzymas can be used to explain life cycle phenomena. However, he was not able to undertake experiments to prove his theory. His idea was ignored and faded away [42].

Although Béchamp's idea was lost to orthodox medicine for various reasons [41], there have been a series of medical scientists sporadically suggesting the existences of living entities at the subcellular level, which is similar to Béchamp's idea. The first one is Reich, a Norwegian medical scientist, who reports discovery of a small element, presumably one that forms the basic foundation of living organisms. Reich called this the *Bione* [35]. The second is Naessens, a French medical scientist, who also claimed to discover the *Somatid*, literally meaning *a small living element *[43], very similar to Béchamp's *microzyma*. Moreover, Lepeshinskaya, a Soviet biologist, claimed that cells need not be formed from other cells but can be formed from noncellular matter. Lepeshinskaya believed that yolk globules were source for new cells, via fusion, in chicken eggs [44]. 

Following the same line, Bonghan Kim further expanded the idea that a small living element is flowing through a circulatory system. Bonghan Kim found microvesicles, which he named “*Sanals*,” floating in the PVS (in Korean literally meaning *living eggs*). Analysing the movement of these microvesicles, Bonghan Kim reported how the living organism works at the subcellular level. He reported that the sanals interact with one another, forming granules that eventually become cells [4].

In order to reexcavate and confirm the pioneering idea of microvesicles at the subcellular level, Lee et al. provided evidence that the fusion of eDNA microvesicles eventually forms a cell-like structure from the fertilizing eggs and the PVS of rat and mice, although the cell-like structure was not proven to be a real cell with the full range of living cell functions [7]. Lee et al. [37] hypothesize that if cell fusion is the basis of life formation, the fusion of eDNA microvesicles, named “microzymas” and “sanal,” should also occur at the subcellular level [37].

## 5. Concluding Remarks: Toward a Theory of the PVS

In this paper, synthesizing the ideas of the pioneers, such as Béchamp and Kim, and the observations from recent research outcomes, we suggest a hypothesis that the PVS is a circulatory network system where microparticles, mainly eDNA microvesicles, interact to form and break down cell structures in response to the biological conditions at the subcellular level [9]. This idea of the PVS is integrating the idea of the Bonghan Kim's Bonghan Ducts, sanals, and Béchamp's microzymas. 

Meanwhile, the exact structural boundary of the PVS is still unknown. Hence, we propose two competing hypotheses regarding the structural boundary of the PVS. One hypothesis is that the PVS is a centralized circulatory system enabling the subcellular level activities within the living being. The other hypothesis is that the PVS is a distribution network bounded by the anatomical structure in which it lies. If this is true, the PVS can be a system that can explain a living organ's response being limited to certain areas on the body.

In addition, we hypothesize that the structural density of the PVS can be limited by the evolutionary level of an animal. In other words, the density of the PVS can be high for the larger animals, compared to the smaller ones. Hence, the function of PVS can be more complex and sensitive to larger animals. Finally, we present the hypothesis that fusion and division of cell structures occur throughout life at the subcellular level [35], spontaneously to maintain the balance of conditions in a creature all through its life cycle.

The PVS has a high potential to be a seminal discovery, although it has been ignored for decades. Proving the functions and demonstrating the complete structure of the PVS, as it operates through fusion and division of cell structure based on cell-free DNA vesicles [9], challenges established views held in the modern medical community. 

Although the anatomical structure and physiological function of the PVS are yet to be proven, the PVS has potential to complement the deficits in modern medicine, dealing with unknown disease causations, including those of chronic pain and cancer. Eventually, the PVS has the potential to reduce medical costs by providing a new arena of complementary or alternative medicine, based around knowledge of the functions and features of the PVS.

## References

[1] Kim BH (1963). On the Kyungrak system. *Journal of the Academy of Medical Sciences of the Democratic People's Republic of Korea*.

[2] Kim BH (1965). The Kyungrak system. *Journal of Jo Sun Medicine*.

[3] Kim BH (1965). Sanal theory. *Journal of Jo Sun Medicine*.

[4] Kim BH (1965). Sanals and hematopoiesis. *Journal of Jo Sun Medicine*.

[5] Soh KS, Soh KS, Kang KA, Harrison DK (2011). Current state of research on the primo vascular system. *The Primo Vascular System, Its Role in Cancer and Regeneration*.

[6] Harrison DK, Vaupel P, Soh KS, Kang KA, Harrison DK (2011). The primo vascular system: facts, open questions, and future perspectives. *The Primo Vascular System, Its Role in Cancer and Regeneration*.

[7] Lee B-C, Lee HS, Yun JE, Kim HA (2013). Evidence for the fusion of extracellular vesicles with/without DNA to form specific structures in fertilized chicken eggs, mice and rats. *Micron*.

[8] Lee SY, Lee B-C, Soh KS, Jhon G, Soh KS, Kang KA, Harrison DK (2001). Development of the putative primo vascular system before the formation of vitelline vessels in chick embryos. *The Primo Vascular System, Its Role in Cancer and Regeneration*.

[9] Lee B-C A hypothesis for a hidden circulation system withcell-free DNA molecules, microvesicles and microparticles: the primo vascular system (Bonghan system), a putative acupuncture meridian system.

[10] Lee B-C, Soh K-S (2008). Contrast-enhancing optical method to observe a Bonghan duct floating inside a lymph vessel of a rabbit. *Lymphology*.

[11] Lee B-C, Yoo JS, Baik KY, Kim KW, Soh K-S (2005). Novel threadlike structures (Bonghan ducts) inside lymphatic vessels of rabbits visualized with a Janus Green B staining method. *Anatomical Record B*.

[12] Lee B-C, Kim S, Soh K-S (2008). Novel anatomic structures in the brain and spinal cord of rabbit that may belong to the Bonghan system of potential acupuncture meridians. *Journal of Acupuncture and Meridian Studies*.

[13] Lee B-C, Eom K-H, Soh K-S (2010). Primo-vessels and primo-nodes in rat brain, spine and sciatic nerve. *Journal of Acupuncture and Meridian Studies*.

[14] Lee B-C, Kim HB, Sung B, Soh KS, Kang KA, Harrison DK (2011). Structure of the sinus in the primo vessel inside the bovine cardiac chambers. *The Primo Vascular System, Its Role in Cancer and Regeneration*.

[15] Lee B-C, Kim HB, Sung B (2011). Network of endocardial vessels. *Cardiology*.

[16] Jia Z, Soh KS, Zhou Q, Bo D, Wenhui Y, Soh KS, Kang KA, Harrison DK (2011). Observation of the primo vascular system on the fascia of dogs. *The Primo Vascular System, Its Role in Cancer and Regeneration*.

[17] Hossein AM, Tian YY, Huang T, Zhang YQ, Che YZ, Zhang WB, Soh KS, Kang KA, Harrison DK (2011). Finding a novel threadlike structure on the intra-abdominal organ surface of small pigs by using in-vivo tripan blue staining. *The Primo Vascular System, Its Role in Cancer and Regeneration*.

[18] Stefanov M (2012). Critical review and comments on B. H. Kim’s work on the primo vascular system. *Journal of Acupuncture Meridian Studies*.

[19] Lee B-C, Yoo JS, Ogay V (2007). Electron microscopic study of novel threadlike structures on the surfaces of mammalian organs. *Microscopy Research and Technique*.

[20] Vodyanov V, Soh KS, Kang KA, Harrison DK (2011). Characterization of primo nodes and vessels by high resolution light microscopy. *The Primo Vascular System, Its Role in Cancer and Regeneration*.

[21] Soo JL, Lee B-C, Chang HN (2008). Proteomic analysis for tissues and liquid from Bonghan ducts on rabbit intestinal surfaces. *Journal of Acupuncture and Meridian Studies*.

[22] Lee B-C, Bae K-H, Jhon G-J, Soh K-S (2009). Bonghan system as mesenchymal stem cell niches and pathways of macrophages in adipose tissues. *Journal of Acupuncture and Meridian Studies*.

[23] Kwon J, Baik KY, Lee B-C, Soh K-S, Lee NJ, Kang CJ (2007). Scanning probe microscopy study of microcells from the organ surface Bonghan corpuscle. *Applied Physics Letters*.

[24] Ogay V, Baik KY, Lee B-C, Soh K-S (2006). Characterization of DNA-containing granules flowing through the meridian-like system on the internal organs of rabbits. *Acupuncture and Electro-Therapeutics Research*.

[25] Kim J, Ogay V, Lee B-C (2008). Catecholamine-producing novel endocrine organ: Bonghan system. *Medical Acupuncture*.

[26] Ogay V, Min SK, Hyo JS, Cheon JC, Soh K-S (2008). Catecholamine-storing cells at acupuncture points of rabbits. *Journal of Acupuncture and Meridian Studies*.

[27] Stefanov M, Kim J (2012). Primo vascular system as a new morphofunctional integrated system. *Journal of Acupuncture Meridian Studies*.

[28] Lee CH, Yoo JS, Kim HH, Kwon J, Soh KS Flow of Nanoparticles inside organs-surface Bonghan ducts.

[29] Sung B, Kim MS, Lee B-C (2008). Measurement of flow speed in the channels of novel threadlike structures on the surfaces of mammalian organs. *Naturwissenschaften*.

[30] Daras JC, Albaredo P, deVernejoul P (1993). Nuclear medicine investigations of transmission of acupuncture information. *Acupuncture in Medicine*.

[31] Stefanov M (2012). Critical review and comments on BH Kim’s work on the primo vascular system. *Journal of Acupuncture Meridian Studies*.

[32] Bechamp A (1912). *The Blood and Its Third Element*.

[33] Lee C, Seol S-K, Lee B-C, Hong Y-K, Je J-H, Soh K-S (2006). Alcian blue staining method to visualize Bonghan threads inside large caliber lymphatic vessels and X-ray microtomography to reveal their microchannels. *Lymphatic Research and Biology*.

[34] Soh K-S (2004). Bonghan duct and acupuncture meridian as optical channel of biophoton. *Journal of the Korean Physical Society*.

[35] Reich W (1979). *The Bion Experiments*.

[36] Lee B-C, Kang DI, Soh KS (2012). *Budding Primo Microcells (Sanals) in A Culture Medium with Fertilized Egg Albumen and RPMI Medium*.

[37] Lee B-C, Lee HS, Kang DI (2012). Growth of extracellular vesicles into cell-like structures in fertilized chick eggs: hypothesis for a mitosis-free alternative path-way. *Journal of Acupuncture Meridian Studies*.

[38] Simpson RJ, Mathivanan S (2012). Extracellular microvesicles: the need for internationally recognised nomenclature and stringent purification criteria. *Journal of Proteomics and Bioinformatics*.

[39] Balaj L, Lessard R, Dai L (2011). Tumour microvesicles contain retrotransposon elements and amplified oncogene sequences. *Nature Communications*.

[40] Ratajczak J, Miekus K, Kucia M (2006). Embryonic stem cell-derived microvesicles reprogram hematopoietic progenitors: evidence for horizontal transfer of mRNA and protein delivery. *Leukemia*.

[41] Hume D (1932). *Bechamp or Pasteur: A Lost Chapter in the History of Biology*.

[42] Machester KL (2001). Antoine Béchamp: père de la biologie, Oui ou non?. *Endaevour*.

[43] Davies R (1991). The story of Gaston Naessens. *Vision*.

[44] Lepeshinskaya OB (1954). *The Origin of Cells From Living Substance*.

